# A dual expression plasmid with Microcin B17 compatible with both prokaryotic and mammalian systems

**DOI:** 10.1016/j.mex.2024.103135

**Published:** 2024-12-26

**Authors:** Agnieszka M. Murakami, Katsuhiro Nagatomo, Hiroshi Koda, Yasutaka Niwa, Manabu Murakami

**Affiliations:** Department of Pharmacology, Hirosaki University Graduate School of Medicine, Hirosaki, Japan

**Keywords:** Bacterial toxin, Protein expression, Fluorescence, Plasmid, DNA recombination

## Abstract

Proteic plasmid addiction systems, such as the control of cell death (Ccd), have been used for efficient plasmid DNA recombination. The CcdB toxin, which has a relatively long sequence of 309 bp, has been the predominant choice for this purpose. However, the need for shorter peptide toxins has emerged. In this study, we evaluated the utility of microcin B17 (MccB17), a peptide consisting of 43 amino acids, in promoting DNA recombination within pgMAX-II, a dual expression plasmid for both prokaryotic and mammalian systems. The insertion of the α-peptide gene from lacZ (α-complementation) demonstrated highly efficient cloning of external DNA in the pgMAX-II/MccB17 plasmid. In both *E. coli* and mammalian cells, the pgMAX-II/MccB17 plasmid effectively facilitated the expression of the DsRed fluorescent protein gene. The results indicate that the novel pgMAX-II/MccB17 plasmid supports efficient and straightforward subcloning of external genes, achieving dual expression in both prokaryotic (*E. coli*) and mammalian systems. This suggests its broad applicability as a versatile dual-expression plasmid.

• The short toxin peptide gene, MccB17, became available.
• MccB17 showed potential for efficient DNA recombination similar to CcdB.
• Using MccB17, we successfully established a dual expression plasmid that functions effectively in both prokaryotic and mammalian cells.

## Graphical abstract

**Figure:**
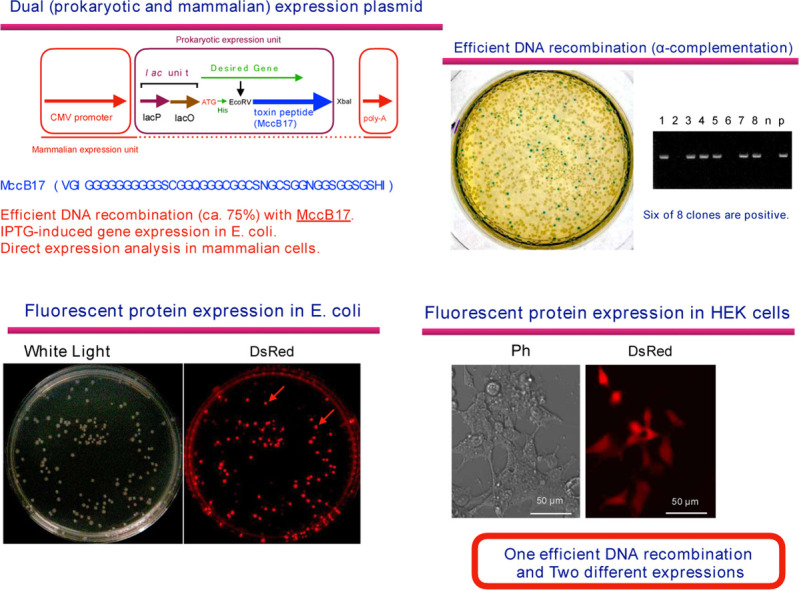

Specifications table

**Table:** 

Subject area:	Pharmacology, Toxicology and Pharmaceutical Science
More specific subject area:	*Cloning and gene expression analysis*
Name of your method:	**pgMAX-II/HccB17; A dual (prokaryotic and mammalian) expression plasmid with Microcin B17**
Name and reference of original method:	pgMAX-II expression plasmid *(*PNAS Nexus 2023, 2, 1–3*)*
Resource availability:	Artificial gene could be made by PCR

## Background

Cloning a single gene and analyzing its functional expression is a well-established technique. In 1994, Bernard et al. developed an efficient subcloning plasmid system that enabled direct positive selection of recombinant DNA through disruption of the lethal control of cell death (Ccd) gene (309 bp) derived from *E. coli* [[1], [2], [3]]. The CcdB toxin, composed of 103 amino acids, operates by trapping bacterial topoisomerase II (DNA gyrase) in a cleaved complex [4,5]. Insertion of a target gene into the CcdB gene disrupts toxin activity, allowing for bacterial growth and colony formation [3]. This application of CcdB in a cloning plasmid enabled effective subcloning of a desired gene. However, further DNA recombination of the cloned gene into an expression plasmid vector is necessary for functional expression analysis.

In 2019, we developed a dual (prokaryotic and mammalian) expression plasmid, pgMAX, which facilitated the direct expression of a target gene in *E. coli* with a simple, single ligation step for efficient gene cloning [6]. Additionally, constructing a mammalian expression vector became straightforward with the removal of the prokaryotic promoter using two different 8-cutter restriction enzymes (SwaI and PmeI), which generate blunt ends for re-ligation with standard DNA ligase. In 2023, we advanced this system with the development of pgMAX-II, a novel plasmid capable of direct expression in both prokaryotic and mammalian cells [7]. In both systems, efficient cloning of external genes relies on the toxic activity of CcdB gene. In contrast, the microbial toxin microcin B17 (MccB17) is a 43-amino-acid antibacterial peptide derived from *E. coli* strains carrying the plasmid-borne mccB17 operon [8]. MccB17 stabilizes the DNA gyrase–DNA cleavage complexes, similar to other topoisomerase II poisons such as CcdB or quinolones. Since Bernard's introduction of CcdB for efficient DNA recombination, CcdB has been the predominant choice, while MccB17 has remained largely unexplored for this application.

In this study, we evaluated the potential of the MccB17 gene, an antibacterial peptide from *E. coli* that stabilizes the transient DNA gyrase–DNA cleavage complex, within the pgMAX expression system [8].

## Method details

**Fig. 1:**
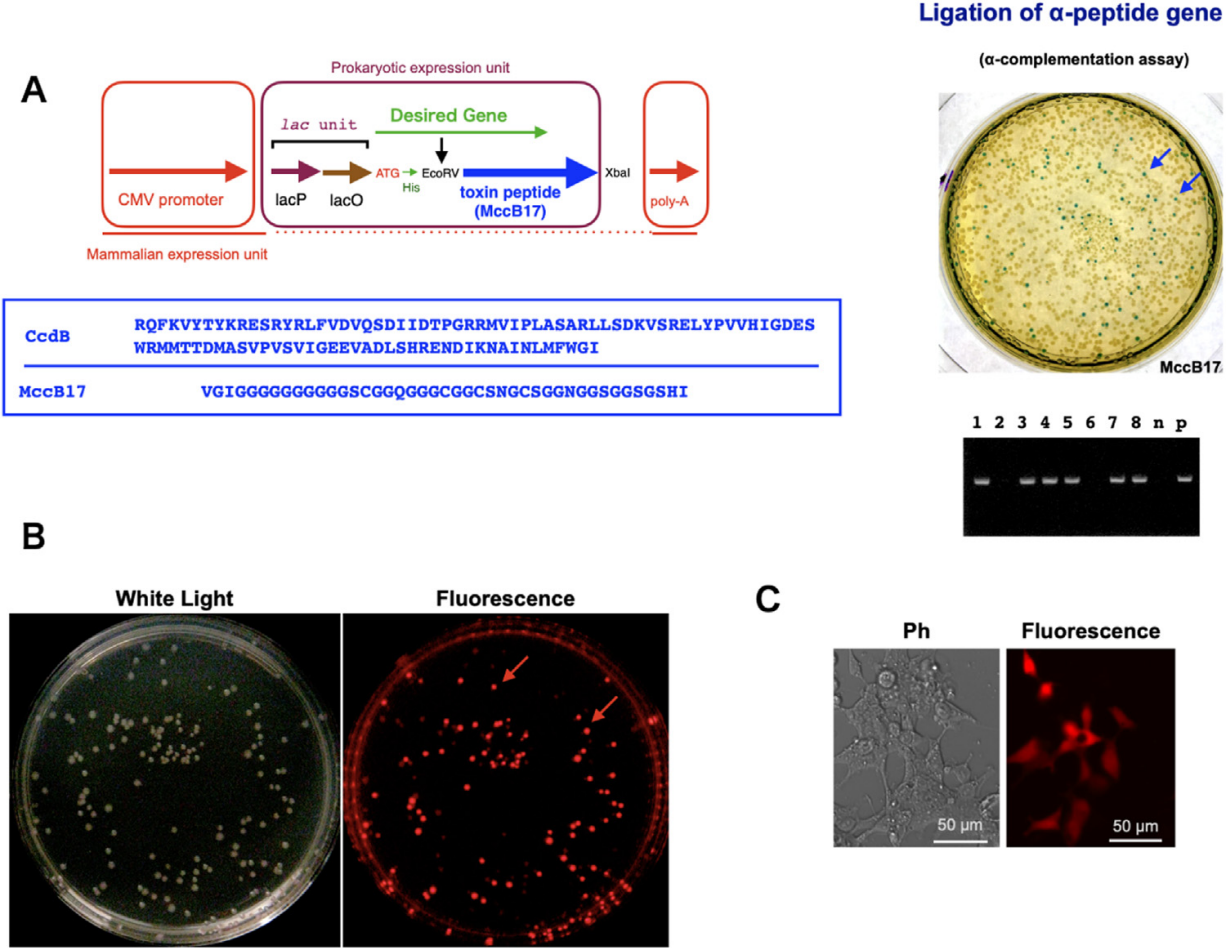
**A. Diagram of the pgMAX-II/(His)/MccB17 dual expression system.** The pgMAX-II plasmid system contains two promoters: a CMV promoter for mammalian expression and a *lac* promoter (*Lac P.*) and operator (*Lac O.*) for prokaryotic expression. The MccB17 toxin sequence was inserted between *EcoRV* and *XbaI*. The target gene (green arrow) is inserted at the *EcoRV* site, resulting in a chimeric fusion protein that includes a poly-histidine tag sequence (His: in green) and toxin gene (blue arrow). The mammalian expression unit, comprising the CMV promoter and poly-A tail, is also shown (in red). (For interpretation of the references to colour in this figure legend, the reader is referred to the web version of this article.)
• Methods
• DNA recombination was performed according to a modified standard method [9]. In brief, PCR was performed to construct the plasmid. The amino acid sequences of CcdB and MccB17 are presented in Fig. 1A [8]. MccB17 peptide toxin gene without leader peptide sequence was inserted between the EcoRV and XbaI restriction sites. The conditions for PCR using high-fidelity Pfu DNA polymerase (Agilent Technologies, Santa Clara, CA, USA) were empirically modified (25 cycles of denaturation at 98 °C for 10 s, annealing at the calculated temperature [∼50 °C] for 30 s, and extension at 72 °C for 30 s). The amplified PCR products were digested with EcoRV and XbaI at 37 °C for 45 min and separated with electrophoresis (1.5 % agarose-gel). DNA fragment was purified using a gel extraction kit with a standard ion-exchange column (Macherey-Nagel GmbH, Duren, Germany). Purified DNA fragment was ligated into EcoRV-XbaI sites in pgMAX with DNA ligase (Takara Bio Inc., Kusatsu, Japan) at 16 °C for 30 min.
• Commercially available Turbo Competent E. coli cells (New England Biolabs, Inc., Ipswich, MA, USA) were used for transformation. Competent cells were developed using the K12 strain with the following genotype: [F’ proA^+^*B*^+^ lacI^q^ ∆lacZM15 / fhuA2 ∆(lac-proAB) glnV galK16 galE15 R(zgb-210::Tn10)Tet^S^ endA1 thi-1 ∆(hsdS-mcrB)5]. Ligated DNA was incubated with 0.1 mL competent cells at 4 °C for 25 min, then 0.9 mL LB medium was added, and incubated by shaking (200 rpm) for 1 h at 37 °C. After 1 h incubation, sample was plated on LB agar supplemented with amp. After 16 h, single colony was inoculated in LB medium (4 ml) containing amp (150 µg/mL) and incubated by shaking (200 rpm) for 12 h at 37 °C. Bacterial cells were harvested by centrifugation (6800 × *g*) for 1 min and plasmid DNA was purified with ion-exchange column of the FastGene Plasmid Mini Kit (Nippon Genetics Europe GmbH, Duren, Germany).

• Materials and methods
• Plasmid construction
• The pgMAX-II plasmid, which contains the lac promoter and lac operator, was used for plasmid construction (Fig. 1A) [6,7]. As previously reported, pgMAX-II contained a CMV promoter and a poly-A sequence, facilitating direct expression analysis in both prokaryotic (E. coli) and mammalian cells without additional DNA recombination [7]. MccB17 polypeptide was prepared by PCR-based amplification using oligo DNA primers [8] (Supplementary Table 1).

## Method validation

### Subcloning and fluorescent protein expression analysis

#### α-complementation assay

To validate DNA recombination and protein expression, a PCR-amplified α-peptide sequence of the lacZ (β-galactosidase) gene was inserted between the blunt-end sites of *EcoRV* (Fig. 1A). The host *E. coli* strain (K12) carries the lacZ deletion mutation (lacZΔM15), which produces the ω-peptide [10]. Following ligation and transformation, the recombinant clones were grown on LB agar containing ampicillin, X-gal, and isopropyl-β-d-thiogalactoside (IPTG) to induce the *lac* operon. After 16 h, the numbers of blue (α-complementation) and white (antisense-directed ligation or absence of DNA fragment insertion) colonies were evaluated [7].

If the DNA is inserted in the sense direction and in-frame with the internal toxin peptide gene, a chimeric fusion peptide composed of α-peptide and toxin DNA is expressed, leading to the formation of blue colonies due to reduced toxin activity. In cases where the toxin has a low inhibitory effect on gyrase, most colonies will appear white due to the absence of the α-peptide coding sequence resulting from self-ligation.

If the toxin inhibits gyrase, the toxin expression will be induced by IPTG in a self-ligated plasmid, inhibiting DNA replication in *E. coli* and preventing colony formation. Therefore, only *E. coli* with a recombinant plasmid containing the α-peptide coding sequence (inserted in either the sense or antisense direction with the *lac* promoter) will form colonies [10]. When the α-peptide coding sequence is inserted in the sense direction with the toxin gene and shares the same reading frame, a chimeric fusion peptide of the α-peptide and the toxin is expressed. This fusion peptide exhibits a mild inhibitory effect on gyrase, allowing *E. coli* to proliferate and form blue colonies [10].

The 43-amino-acid MccB17 demonstrated strong efficacy, as confirmed by PCR screening (Fig. 1A, right panel, with six out of eight colonies containing the inserted DNA) and the α–complementation assay (27.3 % blue colonies), indicating effective toxicity. This result was comparable to that achieved using CcdB (25.4 % blue colonies, Supplementary Fig. 1B).

A blunt-end DsRed2 DNA fragment was amplified using Pfu DNA polymerase with DsRed2-specific oligonucleotides (DsRed2for: 5′-AAAGCTAGCATGGCCTCCTCCGAGAACGTCATCA-3′; DsRe-d2rev: 5′-AAAGAATTCAGATCTCAGGAACAGGTGGTG-3′). The PCR-amplified fluorescent gene was inserted into the *Eco*RV site of the IPTG expression unit. After ligation and transformation, the recombinant clones were plated on LB agar containing ampicillin and IPTG for *lac* operon induction.

We ligated the DsRed gene to assess whether this novel expression plasmid for MccB17 could be applied to other genes.

The PCR-amplified DsRed2 fragment (∼700 bp) was inserted it into the *EcoRV* site of the pgMAX-II/MccB17. After 16 h of incubation, the colonies were observed under green light (excitation wavelength: 563 nm) through a filter set (emission wavelength: 582 nm). Colonies containing the DsRed2 insert in the sense direction downstream of the *lac* promoter exhibited bright red fluorescence in the three constructs (Fig. 1B, red arrows).

### Cell culture and transfection of HEK293 cells

Cell culture and lipofection were performed using standard protocols [7]. HEK293 cells (ATCC CRL 1573; ATCC, Manassas, VA, USA) were cultured in Dulbecco's Modified Eagle's Medium supplemented with 10 % fetal bovine serum. Exponentially growing cells were plated onto 35 mm dishes, and transfection was carried out using commercially prepared lipofectamine (Invitrogen, Carlsbad, CA, USA).

A single DsRed-containing clone was transiently expressed in HEK293 cells. After 48 h of plasmid DNA transfection in HEK293 cells, DsRed fluorescence was observed under a fluorescence microscope (DsRed; excitation wavelength: 563 nm, emission wavelength: 582 nm). The pgMAX-II/DsRed/MccB17 plasmid, which contains the *DsRed* gene inserted at the *Eco*RV site and exhibited bright red fluorescence in *E. coli*, was directly used (without further DNA recombination or deletion of the prokaryotic unit) for transient transfection in HEK293 cells. Bright red fluorescence was observed in HEK293 cells transfected with the pgMAX-II/DsRed/MccB17 plasmid (Fig. 1C, right panel).

Previous cloning plasmids were limited to subcloning the target gene. In contrast, pgMAX-II enables efficient cloning of foreign genes by inducing the CcdB toxin gene with IPTG. Moreover, although at a lower frequency, it allows the expression of the target gene in *E. coli*, meaning that a single-step subcloning can construct an expression vector. The novel pgMAX-II plasmid facilitates target gene expression not only in *E. coli* but also in mammalian cells without the need for genetic recombination. This highlights its potential for functional cDNA expression cloning of desired genes.

In this study, the MccB17 toxin, inserted into pgMAX-II, is significantly shorter at 132 bases, compared to the conventional CcdB, which has a length of 309 bases. Given that commonly used oligonucleotides are ∼60 bases long, the MccB17 sequence can be easily amplified with a few rounds of PCR. This suggests that it could be readily integrated into many existing expression plasmids through relatively simple genetic manipulation.

In general, longer peptides are more prone to nonspecific binding with other proteins. Therefore, short toxins that maintain sufficient activity are particularly valuable. Moreover, in the context of protein-protein interactions, such as antigen-antibody reactions, shorter toxin sequences in chimeric toxins are expected to reduce the likelihood of interactions with peptides.

In conclusion, we evaluated MccB17 as a selectable marker for plasmid DNA recombination using the pgMAX-II system. MccB17 demonstrated external DNA cloning, comparable to CcdB (Fig 1A and Supplementary Fig. 1B). This novel expression plasmid confirmed simple and effective subcloning of external gene, with dual expression capabilities in both prokaryotic (*E. coli*) and mammalian cells.

### *Inset:* amino acid sequences of ccdb and mccb17 genes

#### Blue/white selection of α-complementation (upper panel) and pcr analysis using specific primers for α-complementation (lower panel)

The MccB17 toxin showed a strong effect on α-complementation, with 27.3 % of colonies turning blue (blue arrows). PCR analysis of pgMAX-II/α-peptide gene ligation is shown in the lower panel. Six out of 8 clones contained the desired insert. (n represents the negative control without DNA; p represents the positive control)

**B. Fluorescent protein expression with MccB17 construct.** Representative fluorescence after the ligation of the DsRed gene. Several colonies exhibited red fluorescence (red arrows) under white light (left panel) and under green light with a red filter setting (563 nm excitation and 582 nm emission, right panel).

**C. Direct plasmid expression in human embryonic kidney (HEK) cells.** Phase-contract (pH) and DsRed fluorescence images of pgMAX-II/DsRed/MccB17 transfected HEK cells (right panels).

## Limitations

None.

## Ethics statements

This study was conducted with the approval of the Institutional Review Board of Hirosaki University.

## CRediT authorship contribution statement

**Agnieszka M. Murakami:** Investigation, Methodology, Validation, Writing – original draft. **Katsuhiro Nagatomo:** Investigation, Methodology, Validation. **Hiroshi Koda:** Investigation, Methodology. **Yasutaka Niwa:** Writing – review & editing. **Manabu Murakami:** Conceptualization, Data curation, Funding acquisition, Investigation, Methodology, Project administration, Validation, Writing – original draft, Writing – review & editing.

## Declaration of competing interest

The authors declare that they have no known competing financial interests or personal relationships that could have appeared to influence the work reported in this paper.

## Data Availability

Data will be made available on request.

## Appendix Supplementary materials

Supplementary material *and/or* additional information [OPTIONAL] Image, application 1
