# Supplementary figures and images for: A dual expression plasmid with Microcin B17 compatible with both prokaryotic and mammalian systems
Source: MethodsX. 2024 Dec 26;14:103135. doi: 10.1016/j.mex.2024.103135 (PMC11755071; doi:10.1016/j.mex.2024.103135)

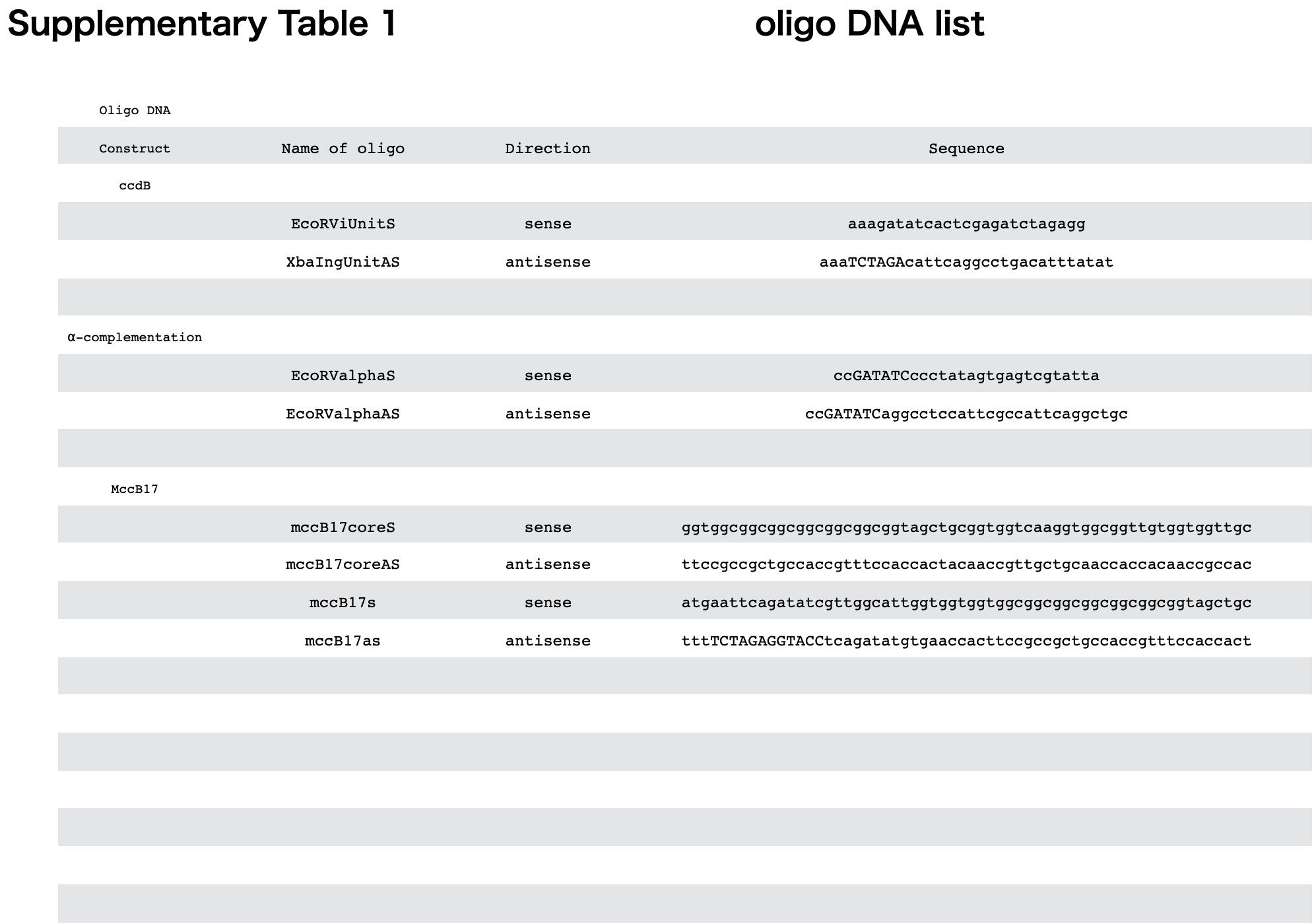


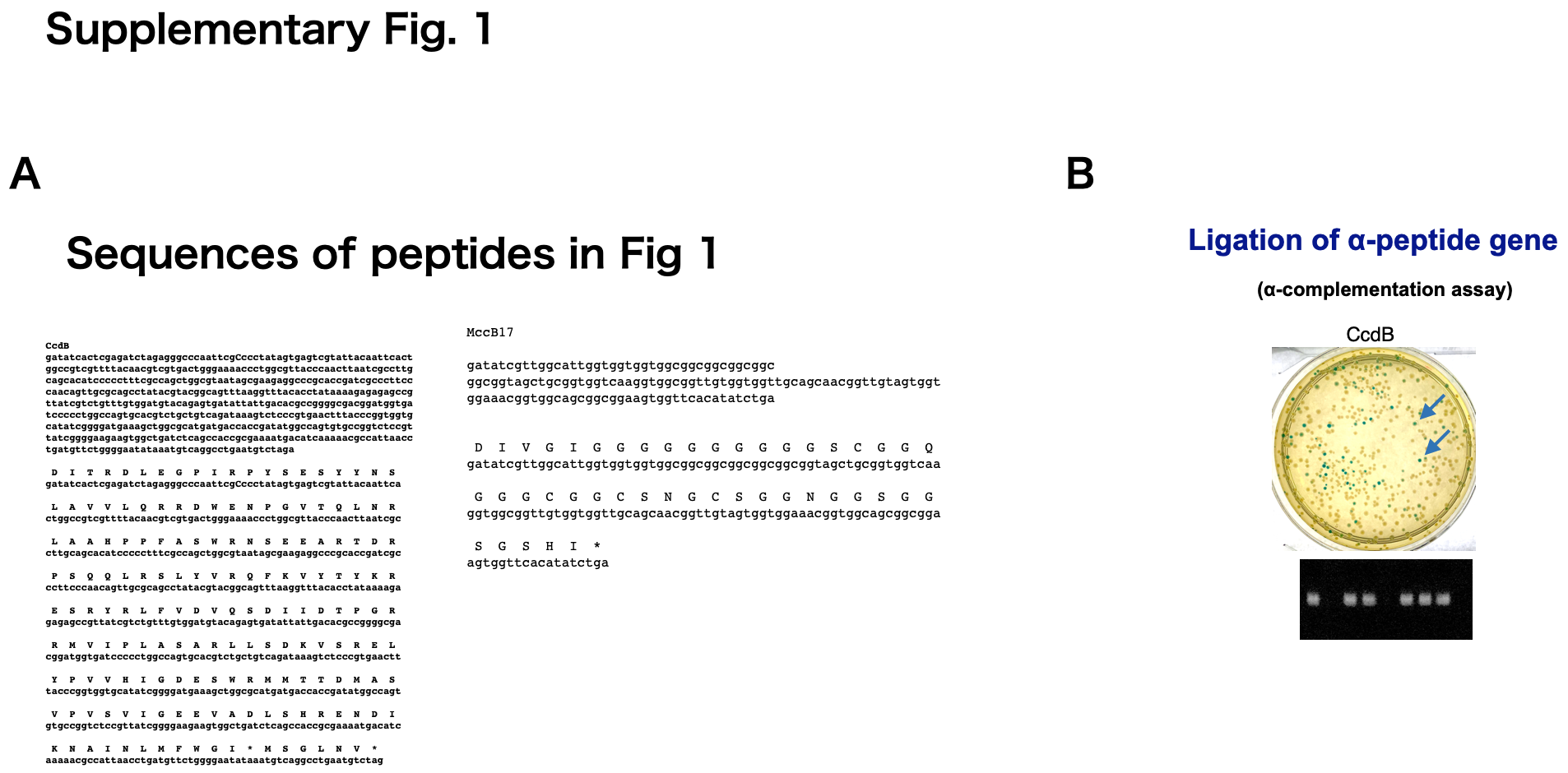


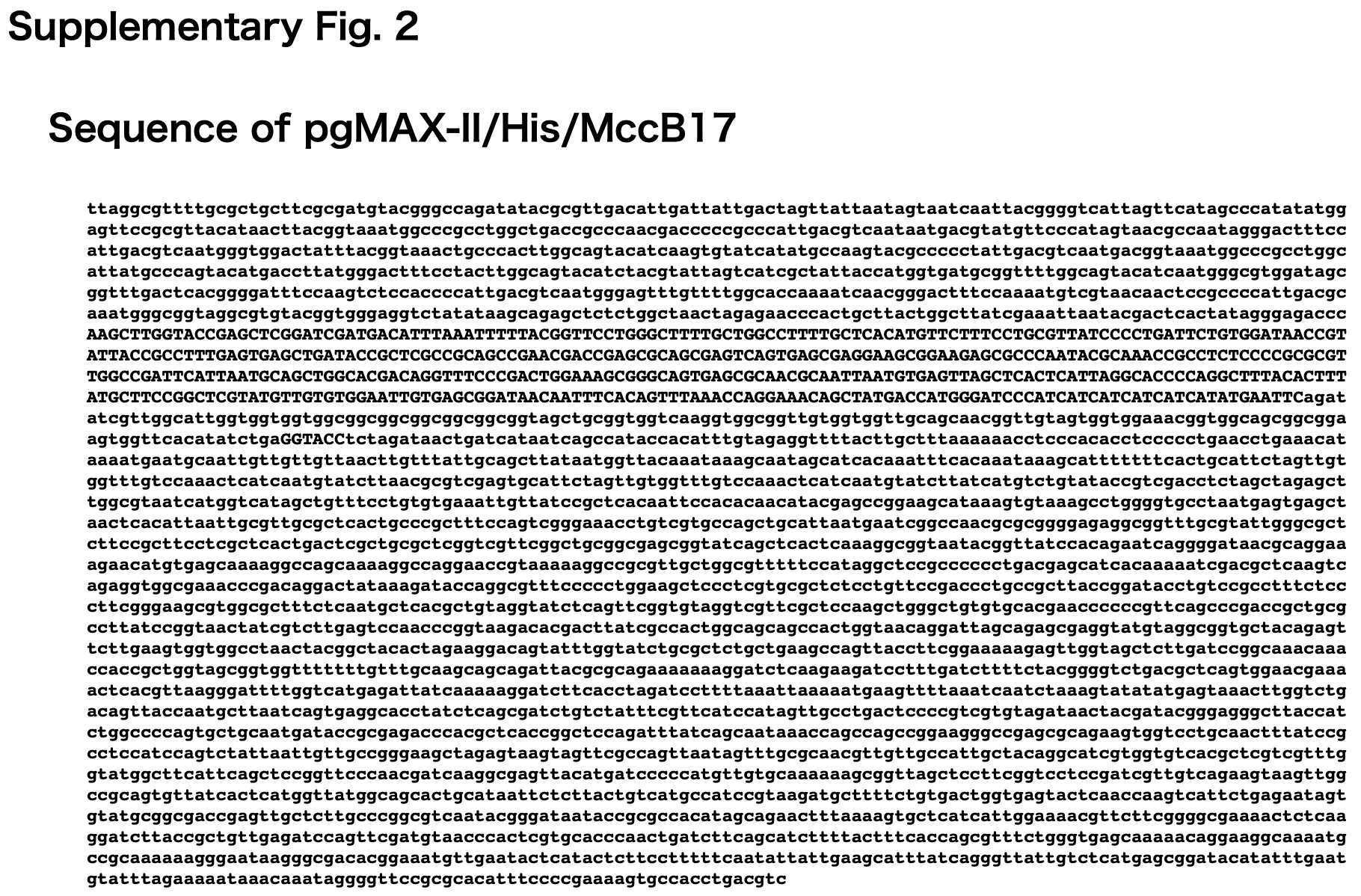


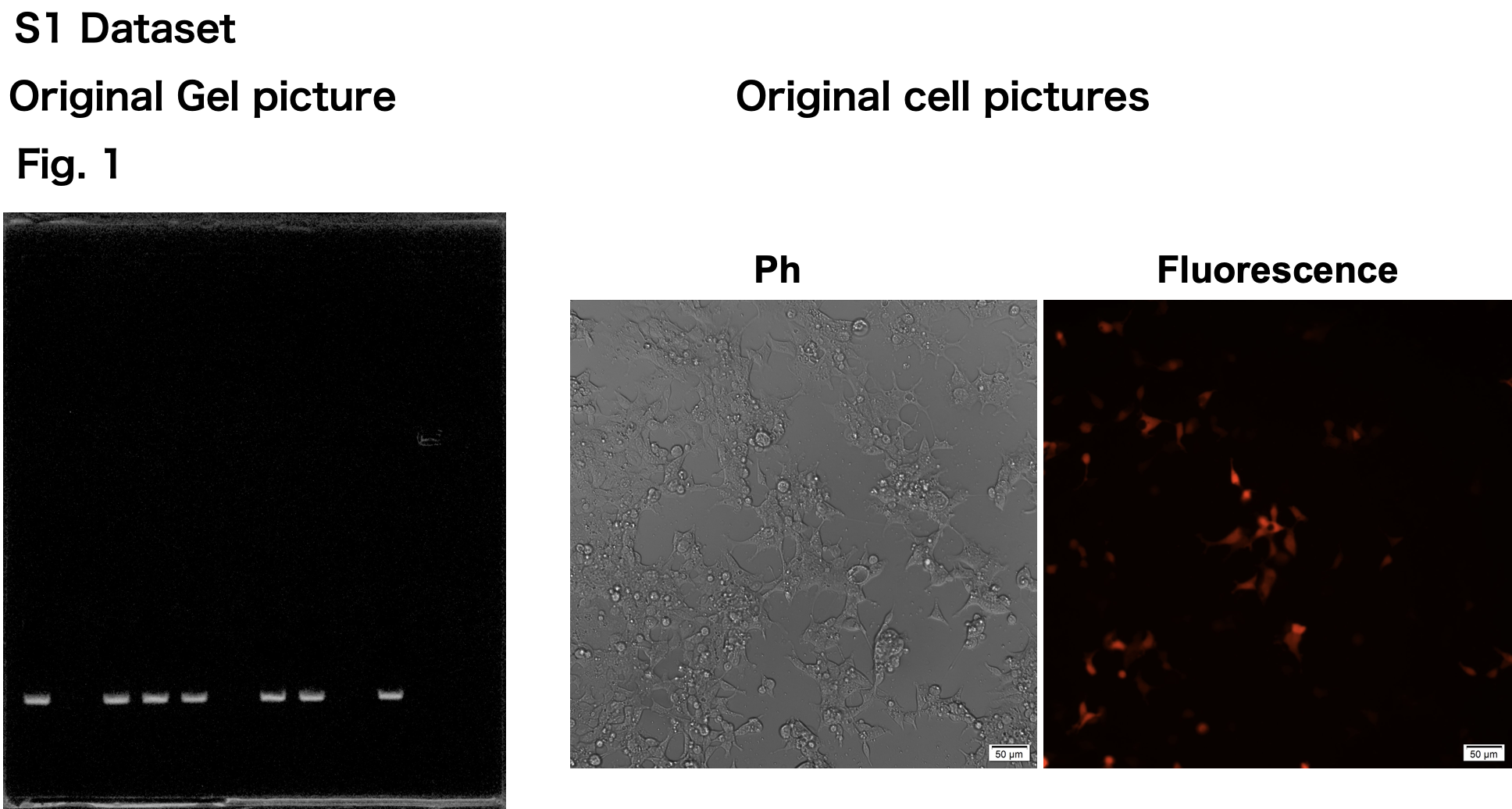

Supplement: Supplementary file 1 — Supplementary material and/or additional information [OPTIONAL] [file mmc1.docx]
